# Advanced Cardiovascular‐Kidney‐Metabolic Risk and Risk‐Factor Control in Patients With Type 2 Diabetes Without Established Cardiovascular Disease

**DOI:** 10.1111/1753-0407.70279

**Published:** 2026-10-19

**Authors:** Hai Nguyen Ngoc Dang, The Trung Le, Anh Kiem Pham, Linh Duy Le, Nam Van Duc Nguyen, Thanh Thien Tran, Thinh Ba Le, Ny Ha Tuyet Le, Nam Van Le, Hau Phuc Tran, Hung Minh Vu, Duong Hung Tran, Bich Ngoc Hoang Thi, Huy Nhu Pham, Hieu Minh Le, Thang Chi Doan, Binh Anh Ho, Tien Anh Hoang, Minh Van Huynh, Thang Viet Luong

**Affiliations:** ^1^ Faculty of Medicine, School of Medicine and Pharmacy Duy Tan University Da Nang Vietnam; ^2^ Menzies Institute for Medical Research, University of Tasmania Hobart Tasmania Australia; ^3^ Da Nang Hospital Da Nang Vietnam; ^4^ Kien Giang General Hospital Rach Gia An Giang Vietnam; ^5^ University of Medicine and Pharmacy, Hue University Hue Vietnam; ^6^ Hue Central Hospital Hue Vietnam; ^7^ Buon Ma Thuot Medical University Tan An Dak Lak Vietnam; ^8^ Internal Cardiology 1, Nghe An Friendship General Hospital Nghe An Vietnam; ^9^ Gia An 115 Hospital Ho Chi Minh City Vietnam; ^10^ Arizona College of Osteopathic Medicine, Midwestern University Glendale Arizona USA; ^11^ Department of Internal Medicine Thai Binh University of Medicine and Pharmacy Hung Yen Vietnam; ^12^ Centre for Teaching and Learning Thai Binh University of Medicine and Pharmacy Hung Yen Vietnam

**Keywords:** cardiovascular‐kidney‐metabolic risk, risk‐factor control, type 2 diabetes mellitus

## Abstract

**Background:**

Cardiovascular‐kidney‐metabolic (CKM) risk stratification may identify patients with type 2 diabetes mellitus (T2DM) who require more intensive primary prevention, but real‐world data from Vietnam are limited. We aimed to describe advanced CKM risk and risk‐factor control in Vietnamese patients with T2DM without established cardiovascular disease (CVD).

**Methods:**

This multicenter cross‐sectional study enrolled 1436 adults with T2DM without CVD in Vietnam. Advanced CKM risk was defined as 10‐year total CVD risk ≥ 20% or eGFR < 60 mL/min/1.73 m^2^. Guideline‐based control of HbA1c, blood pressure, LDL‐C, triglycerides, and non‐HDL‐C was evaluated. Logistic regression assessed associations between advanced CKM risk and target achievement.

**Results:**

Advanced CKM risk was identified in 551 patients (38.4%). Despite a relatively low prevalence of BMI ≥ 25 kg/m^2^ (26.9%), hypertriglyceridemia was the most common metabolic abnormality, affecting 88.0% of participants. Risk‐factor control was generally low, with 33.3% achieving glycemic control, 33.5% blood pressure control, 18.8% LDL‐C control, 12.0% triglyceride control, and 21.1% non‐HDL‐C control. After adjustment, advanced CKM risk was associated with poorer blood pressure control (adjusted OR = 0.466, 95% CI = 0.359–0.603, *p* < 0.001) and triglyceride control (adjusted OR = 0.677, 95% CI = 0.473–0.969, *p* = 0.033), but not with achievement of HbA1c, LDL‐C, non‐HDL‐C, or combined lipid control.

**Conclusions:**

Advanced CKM risk was common among Vietnamese patients with T2DM without established CVD and was consistently associated with poorer blood pressure control. CKM‐based risk assessment may identify high‐risk patients earlier and support guideline‐directed risk‐factor management. Frequent hypertriglyceridemia with relatively low obesity supports a CKM‐based strategy tailored to this metabolic risk pattern.

## Highlight

Advanced cardiovascular‐kidney‐metabolic risk was present in more than one‐third of Vietnamese patients with type 2 diabetes mellitus who do not have recognized cardiovascular disease. This advanced risk was consistently associated with poorer blood pressure control and lower target achievement for lipids.

## Graphical abstract

In this multicenter cross‐sectional study in Vietnam, 1436 adults with type 2 diabetes without established cardiovascular disease were included. Advanced cardiovascular‐kidney‐metabolic (CKM) risk was present in 38.4% of participants. Hypertriglyceridemia affected 88.0%, while the median body mass index was 23.1 kg/m^2^. Only about one‐third achieved glycemic or blood pressure targets, fewer than one‐quarter achieved individual lipid targets, and only 2.8% achieved all three lipid targets. Advanced CKM risk was associated with poorer blood pressure control (aOR = 0.47) and triglyceride control (aOR = 0.68). Overall, the findings show a substantial CKM risk burden and persistent gaps in risk‐factor control. In this population, blood pressure and lipid management may warrant greater attention alongside glycemic control.

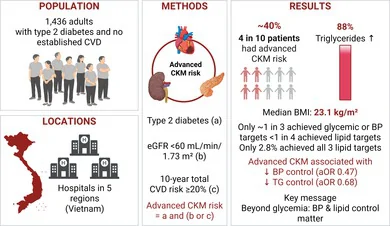

## 1 Introduction

Cardiovascular disease (CVD) is a major cause of morbidity and mortality among patients with type 2 diabetes mellitus (T2DM) [1]. Meanwhile, diabetic kidney disease is one of the most common and clinically important microvascular complications of diabetes, and it also serves as a marker that amplifies the risk of mortality and CVD events [2]. These relationships indicate that T2DM should not be viewed solely as a glycemic disorder but as part of a broader cardiovascular‐kidney‐metabolic (CKM) risk continuum, in which metabolic dysfunction, kidney impairment, and cardiovascular risk interact to worsen prognosis [3]. However, this risk is not uniform among patients with T2DM. Some patients achieve good control of their risk factors, whereas others already have high predicted CVD risk, impaired kidney function, albuminuria, or suboptimal control of blood pressure, lipids, and glycemia [4]. Therefore, in patients with T2DM without clinically established CVD, CKM risk stratification is particularly important for primary prevention, as it helps identify high‐risk individuals before overt CVD events occur.

Accordingly, the CKM framework has been proposed to integrate metabolic, kidney, and CVD risk axes into a unified model for assessment and management [3]. In parallel, several models have been developed to estimate absolute CVD risk within the broader context of CKM health, including risks of atherosclerotic cardiovascular disease (ASCVD), heart failure, and total CVD events [5]. Although these approaches provide an important foundation for integrated risk assessment, their application in clinical practice remains incompletely characterized. In particular, among patients without overt CVD events, data remain limited on the proportion of individuals already classified as high risk and on the extent to which modifiable risk factors are adequately controlled. This evidence gap is especially relevant in Vietnam, where real‐world data on risk stratification remain scarce. Therefore, this study aimed to describe the burden of advanced CKM risk and evaluate the control of key modifiable risk factors among patients with T2DM without clinically established CVD.

## 2 Materials and Methods

### 2.1 Study Design and Study Population

This multicenter, cross‐sectional study was conducted between January 2024 and August 2025 at six hospitals across five provinces and cities in Vietnam. The study protocol was approved by the Hue University of Medicine and Pharmacy Ethics Council (approval number: H2023/014). The selection of participating centers strictly followed the study protocol and adhered to the principles outlined in the Declaration of Helsinki 2013. The participating hospitals included Hue Central Hospital (Hue), Hue University of Medicine and Pharmacy Hospital (Hue), Da Nang General Hospital (Da Nang), and Nghe An General Hospital (Nghe An) in central Vietnam, Thai Binh General Hospital (Hung Yen) in northern Vietnam, and Kien Giang General Hospital (An Giang) in southern Vietnam. Figure 1 shows the geographic distribution by province/city, whereas the participating centers were hospitals located within these provinces/cities.

**FIGURE 1:**
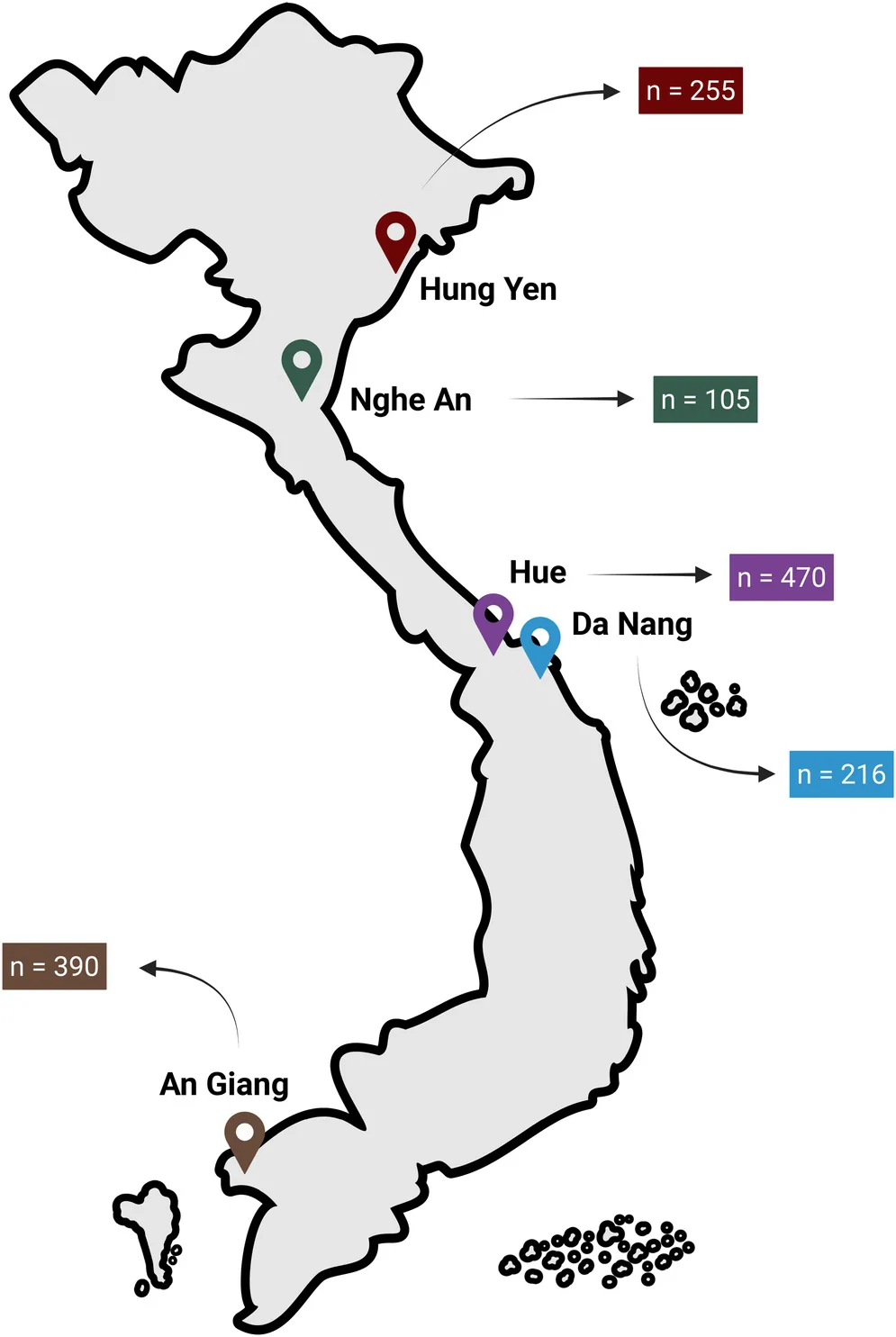
Geographic distribution of study sites and sample sizes of patients with type 2 diabetes mellitus across five provinces and cities in Vietnam.

Eligible participants were patients aged 30–79 years with T2DM diagnosed according to the 2023 American Diabetes Association criteria [6]. Patients were excluded if they declined to participate or were being evaluated for other forms of diabetes, including type 1 diabetes, secondary diabetes, or latent autoimmune diabetes in adults. We also excluded patients with a history of CVD, including stroke, transient ischemic attack, heart failure, coronary artery disease, renal artery disease, or aortic dissection. Pregnant or breastfeeding women were also excluded. These criteria were applied to define a study population suitable for cardiovascular risk assessment using the Predicting Risk of Cardiovascular Disease EVENTs (PREVENT) tool [7].

The minimum required sample size was estimated using Cochran's method for a single proportion. The expected proportion was set at 50%, with a 95% confidence level and an absolute precision of 3%. Based on these assumptions, the minimum required sample size was 1067 participants. After applying the eligibility criteria, a total of 1436 participants from different regions of Vietnam were included in the final analysis.

### 2.2 Data Collection and Study Tools

Clinical data were collected through structured interviews conducted by trained endocrinologists who were independent of the research team. The collected information included age, sex, smoking status, history of CVD, duration of diabetes, hypertension, and current use of antihypertensive medications, statins, or other lipid‐lowering therapies. Systolic and diastolic blood pressure, height, and weight were measured during clinical examination. Height and weight were measured using calibrated equipment with a precision of 0.1 cm and 0.5 kg, respectively. Body mass index (BMI) was calculated as weight in kilograms divided by height in meters squared. Blood pressure measurements were performed according to the recommendations of the American Heart Association [8].

Blood samples were obtained after at least 8 h of fasting. Laboratory parameters included glycated hemoglobin (HbA1c), serum creatinine, total cholesterol (TC), low‐density lipoprotein cholesterol (LDL‐C), high‐density lipoprotein cholesterol (HDL‐C), and triglycerides (TG), all of which were directly measured. Non‐HDL‐C was calculated as TC minus HDL‐C. The estimated glomerular filtration rate (eGFR) was calculated using the Chronic Kidney Disease Epidemiology Collaboration (CKD‐EPI) creatinine equation [9]. Laboratory tests were performed at the clinical laboratories of the participating hospitals. Each participating hospital followed its routine internal calibration and quality‐control procedures.

### 2.3 Assessment of Cardiovascular, Kidney, and Metabolic Risk Domains

The cardiovascular risk domain was assessed using the American Heart Association PREVENT equations. PREVENT estimates 10‐year CVD risk in adults aged 30–79 years without known CVD. In this study, 10‐year total CVD risk was used to define the cardiovascular risk domain. Participants were classified as low risk (< 5%), borderline risk (5% to < 7.5%), intermediate risk (7.5% to < 20%), or high risk (≥ 20%). The calculation used available clinical and laboratory variables incorporated in PREVENT, including age, sex, smoking status, antihypertensive medication use, lipid‐lowering therapy, diabetes status, systolic blood pressure, BMI, TC, HDL‐C, and eGFR [7].

The kidney domain was assessed using eGFR categories. Kidney function was categorized as G1 (eGFR ≥ 90 mL/min/1.73 m^2^), G2 (60–89 mL/min/1.73 m^2^), G3a (45–59 mL/min/1.73 m^2^), G3b (30–44 mL/min/1.73 m^2^), G4 (15–29 mL/min/1.73 m^2^), and G5 (< 15 mL/min/1.73 m^2^). For the primary analysis, reduced kidney function was defined as eGFR < 60 mL/min/1.73 m^2^, corresponding to G3a–G5 [10, 11].

The metabolic risk domain beyond T2DM was defined using prespecified obesity and lipid abnormalities. Hypertriglyceridemia was defined as TG ≥ 1.7 mmol/L, elevated LDL‐C as LDL‐C ≥ 2.6 mmol/L, and high total cholesterol as TC ≥ 5.2 mmol/L [12]. Low HDL‐C was defined as HDL‐C < 1.0 mmol/L in men or < 1.3 mmol/L in women [13]. Elevated non‐HDL‐C was defined as non‐HDL‐C ≥ 3.4 mmol/L [14]. Obesity was defined as BMI ≥ 25 kg/m^2^ according to Asian‐specific BMI criteria [15].

CKM stages were defined with reference to the American Heart Association CKM staging framework [3]. Given the absence of urinary albumin‐to‐creatinine ratio (ACR) measurements, CKM Stage 2 was defined as T2DM without Stage 3 criteria, whereas CKM Stage 3 was operationally defined for this study as high predicted 10‐year total CVD risk by PREVENT (≥ 20%) or reduced kidney function defined by eGFR < 60 mL/min/1.73 m^2^ [16]. For analysis, Stage 2 was classified as non‐advanced CKM risk, whereas Stage 3 was classified as advanced CKM risk.

### 2.4 Risk Factor Control

Risk factor control was assessed using prespecified guideline‐based targets. LDL‐C control was defined as < 1.8 mmol/L and TG control as < 1.7 mmol/L according to the American Diabetes Association Standards of Care in Diabetes [1]. Non‐HDL‐C control was defined as < 2.6 mmol/L according to the 2019 European Society of Cardiology/European Atherosclerosis Society guidelines [17]. Blood pressure control targets were based on the 2020 International Society of Hypertension Global Hypertension Practice Guidelines: blood pressure < 130/80 mmHg for individuals younger than 65 years, and blood pressure < 140/90 mmHg for those aged 65 years or older [18]. Glycemic control was defined as HbA1c < 7% (53 mmoL/mol) for most nonpregnant adults according to the American Diabetes Association Standards of Care in Diabetes [19].

### 2.5 Statistical Analysis

All statistical analyses were performed using the R Project for Statistical Computing version 4.0.3 (R Foundation for Statistical Computing, Vienna, Austria). The normality of continuous variables was assessed using the Shapiro–Wilk test. Categorical variables are presented as frequencies and percentages. Continuous variables are expressed as means with standard deviations or medians with interquartile ranges, as appropriate. Between‐group comparisons were performed using the independent‐samples *t*‐test or the Mann–Whitney *U*‐test for continuous variables and the chi‐squared test or Fisher's exact test for categorical variables. Logistic regression was used to evaluate the association between advanced CKM risk and achievement of risk‐factor control targets. Crude and multivariable‐adjusted models were fitted. The adjusted models included age, sex, BMI, duration of diabetes, and smoking status. In addition, antihypertensive medication use was included in the model for blood pressure control, lipid‐lowering medication use was included in models for lipid‐control outcomes, and both medication variables were included in the model for HbA1c control. Results are presented as odds ratios (ORs) with 95% confidence intervals (CIs). Several sensitivity analyses were performed. First, advanced CKM risk was redefined using high PREVENT risk alone. Second, stricter kidney‐function thresholds were applied, in which advanced CKM risk was defined as high PREVENT risk or eGFR < 45 mL/min/1.73 m^2^ and as high PREVENT risk or eGFR < 30 mL/min/1.73 m^2^. Third, kidney‐only analyses were performed using eGFR < 60 mL/min/1.73 m^2^ and eGFR < 45 mL/min/1.73 m^2^ to partly address potential construct overlap related to PREVENT‐based risk classification. Fourth, lipid‐control analyses were repeated after excluding participants with TG > 4.5 mmol/L. Fifth, blood pressure‐control analyses were repeated using uniform thresholds of < 130/80 and < 140/90 mmHg. Additional analyses were performed to account for site‐level clustering, including cluster‐robust standard errors, hospital and region fixed‐effect models, and mixed‐effects logistic regression with a hospital‐level random intercept. Additional lipid‐control sensitivity analyses were performed using risk‐stratified LDL‐C and non‐HDL‐C targets. To further explore potential construct overlap with systolic blood pressure, an exploratory modified PREVENT‐derived classification was also evaluated after removing systolic blood pressure‐related terms from the linear predictor. Complete‐case analysis was planned for variables required in the main analyses. A two‐sided *p* < 0.05 was considered statistically significant.

## 3 Results

### 3.1 Baseline Characteristics and CKM Risk Profile

A total of 1436 patients with T2DM without established CVD were included. The mean age was 58.0 ± 7.1 years, and 755 patients (52.6%) were male. The median BMI was 23.1 kg/m^2^ [21.3–25.3], and the mean duration of diabetes was 4.4 ± 3.7 years. Current smoking was reported in 366 patients (25.5%); 918 patients (63.9%) were using antihypertensive medication; and 715 patients (49.8%) were using statins or lipid‐lowering therapy. The median PREVENT 10‐year total CVD risk was 15.1% [10.2–20.4], and the median eGFR was 72.9 mL/min/1.73 m^2^ [59.4–91.5] (Table 1).

**TABLE 1:** Baseline characteristics of the study population.

Variable	Overall (*n* = 1436)
Age (years)	58.0 ± 7.1
Male, *n* (%)	755 (52.6)
Weight (kg)	63.6 ± 12.2
Height (cm)	165.3 ± 8.7
BMI (kg/m^2^)	23.1 [21.3–25.3]
Duration of diabetes (years)	4.4 ± 3.7
Age at diabetes diagnosis (years)	53.6 ± 7.9
Current smoker, *n* (%)	366 (25.5)
Using antihypertensive medication, *n* (%)	918 (63.9)
Using statins or lipid‐lowering therapy, *n* (%)	715 (49.8)
Systolic blood pressure (mmHg)	130 [120–140]
Diastolic blood pressure (mmHg)	80.9 ± 17.6
HbA1c (%)	7.8 [6.6–9.4]
Total cholesterol (mmol/L)	5.0 [3.9–5.8]
LDL‐C (mmol/L)	3.0 [2.0–4.0]
HDL‐C (mmol/L)	1.0 [0.9–1.3]
Non‐HDL‐C (mmol/L)	3.7 [2.8–4.6]
Triglycerides (mmol/L)	4.3 [2.5–5.0]
eGFR (mL/min/1.73 m^2^)	72.9 [59.4–91.5]
PREVENT 10‐year total CVD risk (%)	15.1 [10.2–20.4]

*Note:* Values are mean ± SD, median [IQR], or *n* (%).

Abbreviations: BMI, body mass index; CVD, cardiovascular disease; eGFR, estimated glomerular filtration rate; HDL‐C, high‐density lipoprotein cholesterol; LDL‐C, low‐density lipoprotein cholesterol.

The cardiovascular risk domain was dominated by intermediate PREVENT risk (61.8%), followed by high risk (26.5%). In the kidney domain, reduced kidney function, defined as eGFR < 60 mL/min/1.73 m^2^, corresponding to G3a–G5, was present in 370 patients (25.8%). Metabolic abnormalities beyond T2DM were common, with a particularly high prevalence of hypertriglyceridemia (88.0%), followed by elevated LDL‐C (60.2%), elevated non‐HDL‐C (56.5%), and low HDL‐C (51.3%) (Figure 2). Regarding TG categories, 1119 patients (77.9%) had TG > 2.3 mmol/L and 701 (48.8%) had TG > 4.5 mmol/L, while no participant had TG > 11.3 mmol/L (Table S1).

**FIGURE 2:**
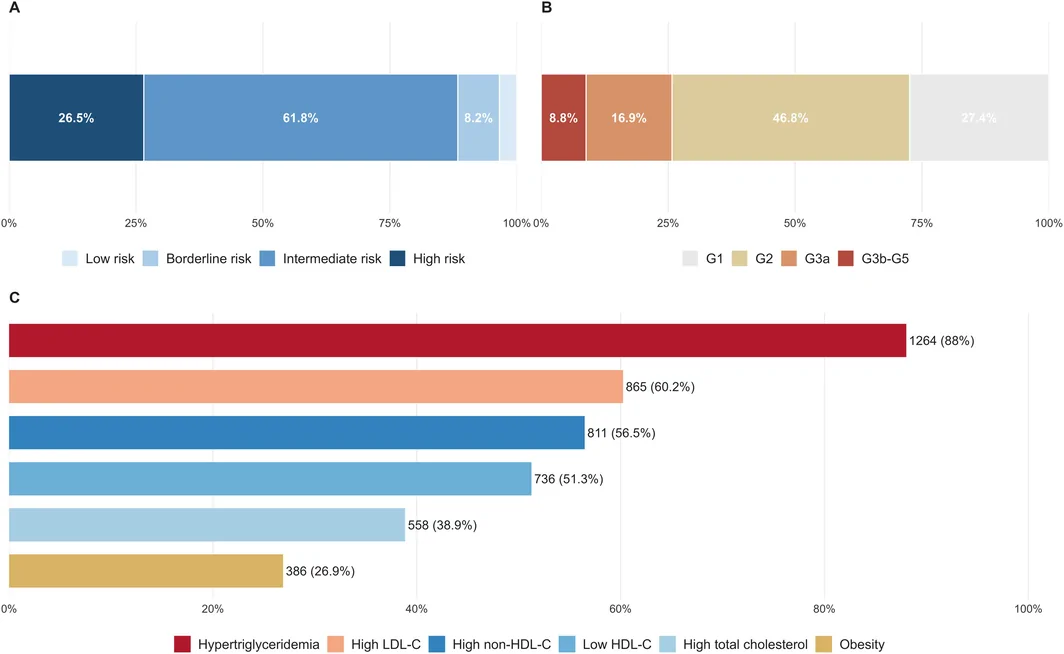
Distribution of cardiovascular, kidney, and metabolic risk domains in patients with type 2 diabetes mellitus without established cardiovascular disease. (A) Cardiovascular risk domain. (B) Kidney domain. (C) Metabolic risk domain beyond type 2 diabetes.

### 3.2 Burden of Advanced CKM Risk

Advanced CKM risk was identified in 551 patients (38.4%), whereas 885 patients (61.6%) were classified as having non‐advanced CKM risk (Figure 3). In the square‐overlap analysis, 181 patients (12.6%) had cardiovascular–metabolic risk without reduced kidney function, 170 patients (11.8%) had kidney–metabolic risk without high PREVENT risk, and 200 patients (13.9%) had concurrent cardiovascular, kidney, and metabolic risk. The remaining 885 patients (61.6%) were classified as metabolic domain only (Figure 4).

**FIGURE 3:**
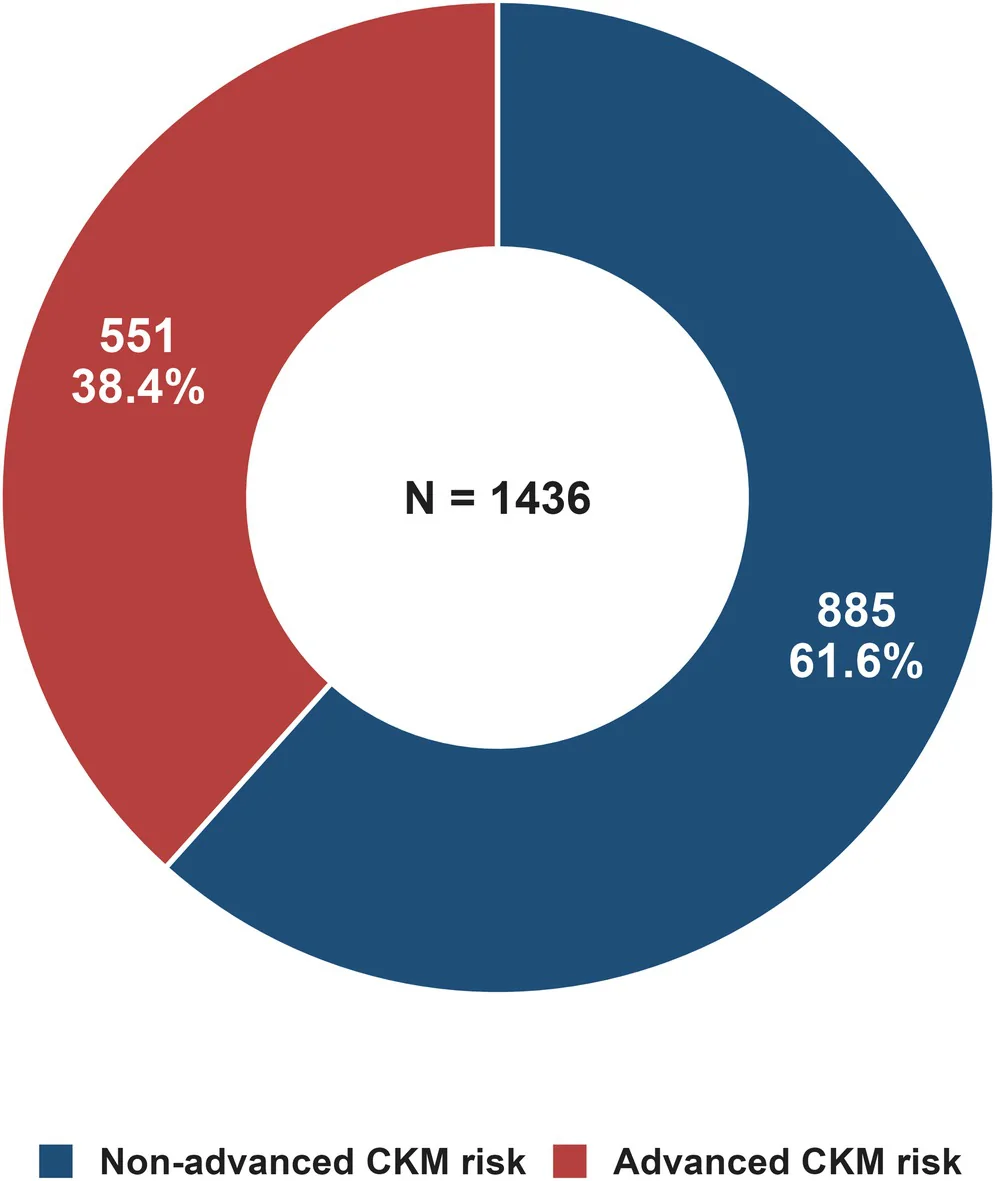
Distribution of advanced cardiovascular‐kidney‐metabolic risk in the study population.

**FIGURE 4:**
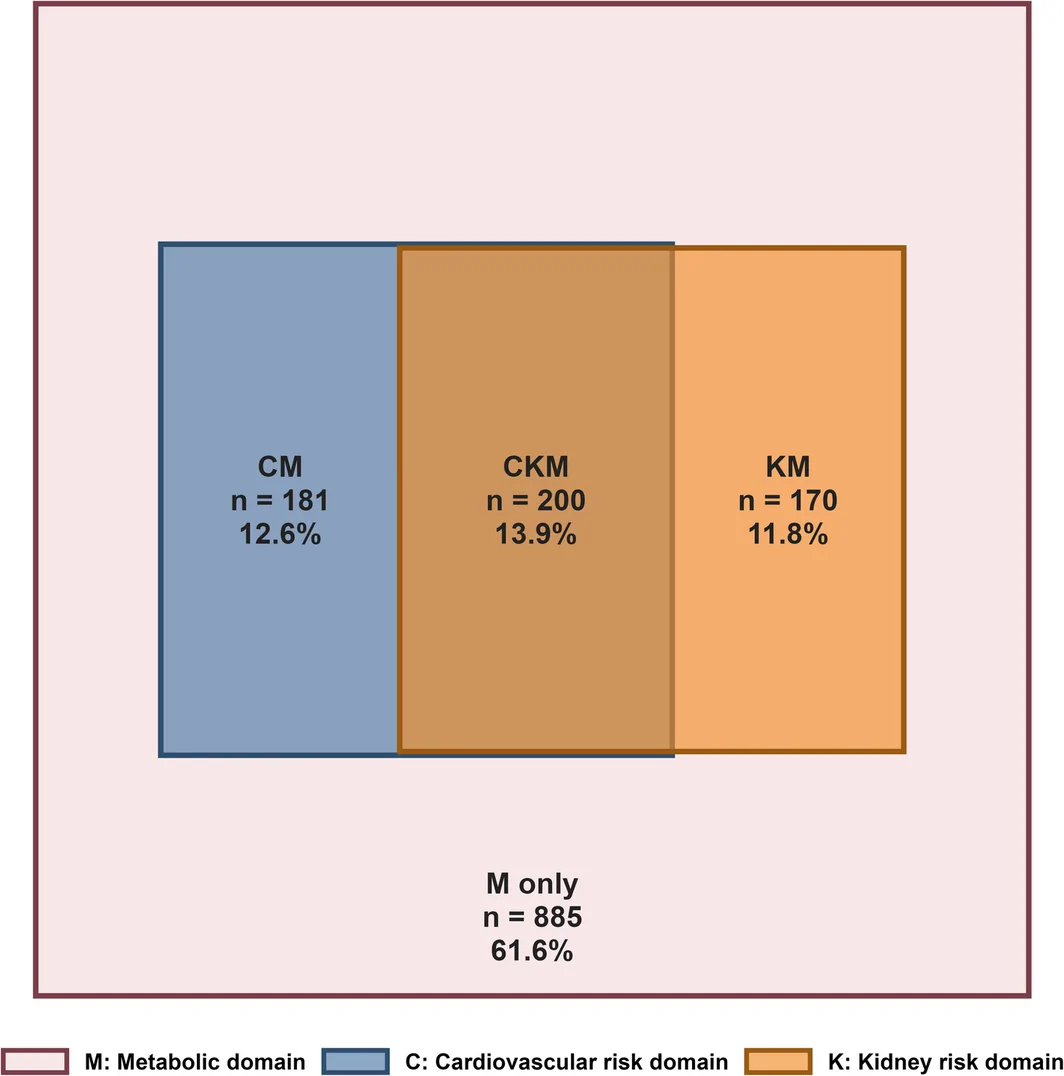
Overlap of cardiovascular and kidney components of advanced cardiovascular‐kidney‐metabolic risk within the metabolic domain.

Compared with patients with non‐advanced CKM risk, those with advanced CKM risk were older and were more likely to be current smokers, receiving antihypertensive medication, and receiving statins. Systolic and diastolic blood pressure were higher in the advanced CKM risk group. In terms of laboratory profile, patients with advanced CKM risk had higher TC, LDL‐C, and non‐HDL‐C levels and lower HDL‐C and eGFR levels. Sex distribution, BMI, duration of diabetes, HbA1c, and TG levels were not significantly different between groups (Table S2).

### 3.3 Control of Modifiable Risk Factors According to Risk Burden

In Table 2, 478 patients (33.3%) achieved HbA1c control, 481 (33.5%) achieved blood pressure control, 270 (18.8%) achieved LDL‐C control, 172 (12.0%) achieved TG control, and 303 (21.1%) achieved non‐HDL‐C control. Compared with the non‐advanced CKM group, the advanced CKM risk group had a numerically lower rate of glycemic control, although the difference did not reach statistical significance (30.5% vs. 35.0%, *p* = 0.076). Blood pressure control was significantly lower in the advanced CKM risk group than in the non‐advanced group in the overall cohort (23.8% vs. 39.5%, *p* < 0.001). Among patients using antihypertensive medication, blood pressure control was achieved in 20.8% of the advanced CKM risk group compared with 33.9% of the non‐advanced group (*p* < 0.001). Differences in LDL‐C, TG, non‐HDL‐C, combined overall control, and combined lipid control were not statistically significant in unadjusted comparisons. To further contextualize these findings across participating centers, center‐level distributions of advanced CKM risk and risk‐factor control are provided in Table S3.

**TABLE 2:** Control of modifiable risk factors according to advanced CKM risk status.

Outcome	Overall	Non‐advanced CKM risk	Advanced CKM risk	*p*
All patients				
Glycemic control	478/1436 (33.3%)	310/885 (35.0%)	168/551 (30.5%)	0.076
Blood pressure control	481/1436 (33.5%)	350/885 (39.5%)	131/551 (23.8%)	< 0.001
LDL‐C control	270/1436 (18.8%)	172/885 (19.4%)	98/551 (17.8%)	0.437
Triglyceride control	172/1436 (12.0%)	115/885 (13.0%)	57/551 (10.3%)	0.133
Non‐HDL‐C control	303/1436 (21.1%)	188/885 (21.2%)	115/551 (20.9%)	0.867
Combined LDL‐C, TG, and non‐HDL‐C control	40/1436 (2.8%)	25/885 (2.8%)	15/551 (2.7%)	0.909
Combined control of HbA1c, BP, LDL‐C, TG, and non‐HDL‐C	3/1436 (0.2%)	3/885 (0.3%)	0/551 (0.0%)	0.290
Patients using antihypertensive medication				
Blood pressure control	256/918 (27.9%)	169/499 (33.9%)	87/419 (20.8%)	< 0.001
Patients using statins or lipid‐lowering therapy				
LDL‐C control	143/715 (20.0%)	86/419 (20.5%)	57/296 (19.3%)	0.676
Triglyceride control	109/715 (15.2%)	69/419 (16.5%)	40/296 (13.5%)	0.279
Non‐HDL‐C control	166/715 (23.2%)	95/419 (22.7%)	71/296 (24.0%)	0.682
Combined LDL‐C, TG, and non‐HDL‐C control	20/715 (2.8%)	13/419 (3.1%)	7/296 (2.4%)	0.556

Abbreviations: BP, blood pressure; CKM, cardiovascular‐kidney‐metabolic; HDL‐C, high‐density lipoprotein cholesterol; LDL‐C, low‐density lipoprotein cholesterol; TG, triglycerides.

### 3.4 Association Between Advanced CKM Risk and Risk‐Factor Control

In logistic regression analysis, advanced CKM risk was associated with lower odds of achieving blood pressure control in both crude and adjusted models. In the adjusted model, the OR was 0.466 (95% CI = 0.359–0.603; *p* < 0.001). Advanced CKM risk was also associated with lower odds of achieving TG control after adjustment (OR = 0.677; 95% CI = 0.473–0.969; *p* = 0.033). Advanced CKM risk was not significantly associated with achievement of HbA1c control, LDL‐C control, non‐HDL‐C control, or combined lipid control after adjustment (Table 3).

**TABLE 3:** Logistic regression analysis of advanced CKM risk and risk‐factor control.

Outcome	Model	OR (95% CI)	*p*
Glycemic control	Crude	0.814 (0.648–1.022)	0.076
	Adjusted	0.835 (0.654–1.067)	0.150
Blood pressure control	Crude	0.477 (0.376–0.605)	< 0.001
	Adjusted	0.466 (0.359–0.603)	< 0.001
LDL‐C control	Crude	0.897 (0.681–1.180)	0.437
	Adjusted	0.864 (0.648–1.152)	0.321
Triglyceride control	Crude	0.773 (0.552–1.082)	0.133
	Adjusted	0.677 (0.473–0.969)	0.033
Non‐HDL‐C control	Crude	0.978 (0.753–1.270)	0.867
	Adjusted	0.922 (0.701–1.214)	0.563
Combined lipid control of LDL‐C, TG, and non‐HDL‐C	Crude	0.963 (0.503–1.843)	0.909
	Adjusted	0.775 (0.391–1.539)	0.467

*Note:* The reference group was non‐advanced CKM risk.

Abbreviations: CI, confidence interval; CKM, cardiovascular‐kidney‐metabolic; HDL‐C, high‐density lipoprotein cholesterol; LDL‐C, low‐density lipoprotein cholesterol; OR, odds ratio; TG, triglycerides.

Sensitivity analyses were performed to assess the robustness of these findings. When advanced risk was redefined using high PREVENT risk alone, lower blood pressure control remained statistically significant after adjustment (OR = 0.407; 95% CI = 0.297–0.560; *p* < 0.001). Similarly, when stricter kidney‐function thresholds were used, defining advanced CKM risk as high PREVENT risk or eGFR < 45 mL/min/1.73 m^2^ and as high PREVENT risk or eGFR < 30 mL/min/1.73 m^2^, the association with lower blood pressure control remained significant (OR = 0.431; 95% CI = 0.316–0.588; *p* < 0.001 and OR = 0.417; 95% CI = 0.304–0.572; *p* < 0.001, respectively). In kidney‐only analyses, eGFR < 60 mL/min/1.73 m^2^ was also associated with lower odds of blood pressure control (OR = 0.618; 95% CI = 0.470–0.814; *p* < 0.001). The association between advanced CKM risk and lower blood pressure control also remained significant when uniform blood pressure targets were applied, including < 130/80 mmHg (OR = 0.421; 95% CI = 0.320–0.554; *p* < 0.001) and < 140/90 mmHg (OR = 0.467; 95% CI = 0.367–0.594; *p* < 0.001). In lipid sensitivity analyses excluding participants with TG > 4.5 mmol/L, advanced CKM risk remained associated with lower odds of TG control (OR = 0.618; 95% CI = 0.424–0.901; *p* = 0.012), whereas associations with LDL‐C, non‐HDL‐C, and combined lipid control were not statistically significant. Detailed sensitivity analyses are shown in Table S4. When blood pressure control was examined according to the source of advanced CKM risk, target achievement was 18.8% in the cardiovascular‐metabolic risk group, 22.5% in the CKM overlap group, 30.6% in the kidney‐metabolic risk group, and 39.5% in the metabolic‐domain‐only group (Table S5). In additional analyses accounting for site‐level clustering, the association between advanced CKM risk and lower blood pressure control remained statistically significant in models using hospital fixed effects and mixed‐effects logistic regression with a hospital‐level random intercept (Table S6). In risk‐stratified lipid‐target sensitivity analyses, advanced CKM risk remained associated with poorer achievement of LDL‐C, TG, and non‐HDL‐C control targets (Table S7). In the exploratory modified PREVENT‐derived analysis after removing systolic blood pressure‐related terms, advanced CKM risk also remained associated with lower blood pressure control and TG control (Table S8).

## 4 Discussion

In this multicenter Vietnamese study of patients with T2DM without established CVD, the burden of cardiovascular, kidney, and metabolic risk was substantial. The vast majority of participants had at least intermediate predicted cardiovascular risk, and approximately one‐quarter had reduced kidney function defined as eGFR < 60 mL/min/1.73 m^2^. This finding is broadly consistent with contemporary risk‐stratification studies showing that many patients with T2DM are not truly low risk in primary prevention [20, 21]. The kidney‐risk burden in our cohort also appeared higher than that reported in some other Asian T2DM cohorts [22]. Dyslipidemia was also highly prevalent, with more than half of patients having hypertriglyceridemia, elevated LDL‐C, or low HDL‐C. This pattern is consistent with previous studies in Asian and Middle Eastern T2DM populations, where dyslipidemia is common and frequently characterized by low HDL‐C, elevated LDL‐C, and high TGs [23, 24]. In contrast, obesity was present in only approximately one‐quarter of the study population. This differs from several Western T2DM cohorts, in which BMI‐defined obesity is often present in more than half of patients [25]. The coexistence of a high CKM risk burden and frequent dyslipidemia despite relatively low obesity prevalence suggests that BMI alone may not fully capture metabolic risk in Vietnamese patients with T2DM. This observation is consistent with evidence that Asian populations may have higher body fat percentages and greater visceral adiposity at a given BMI, allowing adverse metabolic profiles to emerge at lower BMI levels than in Western populations [26].

When these cardiovascular and kidney abnormalities were integrated into the CKM staging framework using high PREVENT risk or eGFR < 60 mL/min/1.73 m^2^, more than one‐third of participants were classified as having advanced CKM risk. Despite this high‐risk profile, treatment‐target achievement remained poor. Only about one‐third achieved glycemic and blood pressure control, while fewer than one‐quarter reached contemporary lipid control targets. These findings highlight a clinically important risk–control gap, in which a substantial proportion of patients had elevated CKM risk but remained suboptimally controlled for major modifiable risk factors. This gap was particularly evident among patients already receiving treatment. Among those using antihypertensive medication, only 27.9% achieved the age‐specific blood pressure target. Similarly, among patients using statins or lipid‐lowering therapy, only 20.0% achieved LDL‐C control, 15.2% achieved TG control, and 23.2% achieved non‐HDL‐C control. More importantly, only 2.8% achieved simultaneous control of LDL‐C, TG, and non‐HDL‐C. These findings suggest a substantial implementation gap in risk‐factor management. Another important observation was the distinctive lipid pattern in this cohort. Although contemporary lipid management frameworks in diabetes remain largely LDL‐C‐centered [1, 17], diabetic dyslipidemia is biologically more complex and is commonly characterized by elevated TG, low HDL‐C, and a predominance of small dense LDL particles [27]. In our cohort, hypertriglyceridemia was by far the most prevalent lipid abnormality, affecting 88.0% of participants, suggesting that TG‐rich dyslipidemia may represent a particularly prominent component of residual metabolic risk among Vietnamese patients with T2DM.

Further analysis according to CKM risk burden showed that patients with advanced CKM risk generally had lower rates of treatment‐target achievement than those with non‐advanced CKM risk. The most pronounced difference was observed for blood pressure control. In the overall cohort, only 23.8% of patients with advanced CKM risk achieved the age‐specific blood pressure target, compared with 39.5% of those with non‐advanced CKM risk. This difference persisted among patients receiving antihypertensive medication, where blood pressure control was achieved in only 20.8% of the advanced CKM risk group compared with 33.9% of the non‐advanced group. Logistic regression analysis further supported this finding, as advanced CKM risk remained strongly associated with lower odds of achieving blood pressure control after adjustment for age, sex, BMI, duration of diabetes, smoking status, and antihypertensive medication use. This association was robust across sensitivity analyses using alternative advanced‐risk definitions, alternative blood pressure targets, kidney‐only analyses, and site‐clustering models. Advanced CKM risk was also associated with lower odds of achieving TG control in the primary adjusted analysis, although this finding was less consistent than the association with blood pressure control. In contrast, advanced CKM risk was not significantly associated with HbA1c control, LDL‐C control, non‐HDL‐C control, or combined lipid control after adjustment in the primary analysis. Overall, poor risk‐factor control was common across the whole T2DM population, while inadequate blood pressure control was the clearest and most consistent management gap among patients with advanced CKM risk.

These findings have several clinical implications. First, patients with T2DM without established CVD should not be assumed to be uniformly low‐risk. Routine CKM‐based risk assessment, incorporating predicted cardiovascular risk and kidney function, may help identify patients who already have advanced risk despite being in a primary prevention setting. Where available, albuminuria assessment should be added to improve kidney‐risk classification. This approach may be particularly relevant in Vietnamese and other Asian populations, where BMI‐defined obesity may not fully capture underlying metabolic risk. Second, the low achievement of treatment targets, particularly among patients already receiving antihypertensive or lipid‐lowering therapy, suggests that treatment exposure alone is insufficient. More structured follow‐up, adherence assessment, treatment intensification, and target‐oriented management are needed. Third, blood pressure control should be prioritized as the most consistent and actionable management gap, especially in patients with advanced CKM risk. At the same time, lipid control remained poor across the whole cohort, and the high prevalence of hypertriglyceridemia suggests that residual metabolic risk in Vietnamese patients with T2DM may extend beyond LDL‐C. Therefore, lipid management should not be limited to LDL‐C lowering alone but should also consider TG‐rich dyslipidemia, lifestyle optimization, glycemic control, and treatment intensification where clinically indicated.

This study has several strengths, including its multicenter design, relatively large sample size, and inclusion of patients from different regions of Vietnam. However, several limitations should be acknowledged. First, the cross‐sectional design precludes causal inference between advanced CKM risk and risk‐factor control. Second, convenience sampling and recruitment from hospital‐based settings may limit generalizability to all Vietnamese patients with T2DM in the community. Third, PREVENT was developed and validated primarily in non‐Vietnamese populations. Therefore, its absolute risk estimates may not be fully calibrated to Vietnamese event rates. In this study, PREVENT was used primarily as a pragmatic tool to operationalize the cardiovascular component of CKM risk rather than to provide definitive locally calibrated absolute‐risk prediction. In addition, because PREVENT includes systolic blood pressure, eGFR, and lipid parameters, residual construct overlap between the exposure definition and several risk‐factor control outcomes cannot be completely excluded. Fourth, urinary ACR was not assessed in this study. Therefore, albuminuria‐related kidney risk could not be captured, and the kidney component of CKM risk was based on eGFR alone. Fifth, laboratory measurements were performed at the clinical laboratories of the participating hospitals rather than at a central laboratory. Although analyses followed routine local laboratory procedures, inter‐laboratory coefficients of variation and detailed assay‐platform harmonization data were not available. Therefore, residual measurement heterogeneity across centers cannot be excluded. Finally, data on medication dose, treatment adherence, lifestyle factors, socioeconomic status, long‐term treatment history, and specific CKM‐relevant therapies such as renin–angiotensin–aldosterone system (RAAS) inhibitors, sodium–glucose cotransporter 2 (SGLT2) inhibitors, and glucagon‐like peptide‐1 (GLP‐1) receptor agonists were unavailable. These unmeasured factors may have contributed to residual confounding and limited our ability to distinguish between greater underlying risk burden and potentially modifiable treatment‐related factors such as adherence, treatment intensity, or therapeutic inertia.

## 5 Conclusion

Among Vietnamese patients with T2DM without established CVD, advanced CKM risk was common and affected more than one‐third of participants. Advanced CKM risk was consistently associated with poorer blood pressure control, while lipid‐target achievement was low across the whole cohort and tended to be less favorable among patients with advanced CKM risk, particularly for TG control in adjusted and sensitivity analyses. These findings highlight the need for integrated CKM‐based risk assessment and more intensive, target‐oriented primary prevention in patients with T2DM.

## Author Contributions

H.N.N.D. and T.V.L. led this project, taking charge of the conceptualization, methodology, data curation, investigation, original draft writing, and review and editing. T.T.L. contributed to methodology, data curation, investigation, original draft writing, and review and editing. A.K.P., L.D.L., N.V.D.N., T.B.L., T.T.T., N.H.T.L., H.M.V., H.N.P., N.V.L., D.H.T., H.P.T., B.N.H.T., and H.M.L. contributed to data curation, formal analysis, and original draft preparation. T.A.H., B.A.H., T.C.D., and M.V.H. provided resources and supervision. All authors reviewed and approved the final version of the manuscript.

## Funding

Open Access funding enabled and organized by CAUL and its Member Institutions.

## Ethics Statement

The study questionnaire and protocol were approved by the Hue University of Medicine and Pharmacy Ethics Council (Approval No. H2023/014). The research was conducted following the guidelines stipulated in the Declaration of Helsinki 2013. All personal information was kept confidential throughout the study.

## Consent

Informed consent was obtained from all participants after providing detailed information about the study, and written consent forms were signed prior to their inclusion.

## Conflicts of Interest

The authors declare no conflicts of interest.

## Supporting information


**Table S1:** Triglyceride distribution of the study population.
**Table S2:** Baseline characteristics according to advanced cardiovascular‐kidney‐metabolic risk status.
**Table S3:** Center‐level distribution of advanced CKM risk and risk‐factor control.
**Table S4:** Sensitivity analyses.
**Table S5:** Blood pressure control according to the source of advanced cardiovascular‐kidney‐metabolic risk.
**Table S6:** Site‐clustering sensitivity analyses for risk‐factor control outcomes.
**Table S7:** Sensitivity analyses using risk‐stratified lipid targets.
**Table S8:** Exploratory sensitivity analysis using a modified PREVENT‐derived classification after removing SBP‐related terms.

## Data Availability

Data supporting this publication will be available through direct request to the first author (Hai Nguyen Ngoc Dang).

## References

[1] American Diabetes Association Professional Practice Committee , “Cardiovascular Disease and Risk Management: Standards of Care in Diabetes—2024,” Diabetes Care 47, no. Supplement_1 (2024): S179–S218, 10.2337/dc24-S010. PMC10725811 38078592

[2] R. Z. Alicic , M. T. Rooney , and K. R. Tuttle , “Diabetic Kidney Disease: Challenges, Progress, and Possibilities,” Clinical Journal of the American Society of Nephrology 12, no. 12 (2017): 2032–2045, 10.2215/CJN.11491116. PMC5718284 28522654

[3] C. E. Ndumele , J. Rangaswami , S. L. Chow , et al., “Cardiovascular‐Kidney‐Metabolic Health: A Presidential Advisory From the American Heart Association,” Circulation 148, no. 20 (2023): 1606–1635, 10.1161/CIR.0000000000001184. 37807924

[4] A. Rawshani , A. Rawshani , S. Franzén , et al., “Risk Factors, Mortality, and Cardiovascular Outcomes in Patients With Type 2 Diabetes,” New England Journal of Medicine 379, no. 7 (2018): 633–644, 10.1056/NEJMoa1800256. 30110583

[5] S. S. Khan , J. Coresh , M. J. Pencina , et al., “Novel Prediction Equations for Absolute Risk Assessment of Total Cardiovascular Disease Incorporating Cardiovascular‐Kidney‐Metabolic Health: A Scientific Statement From the American Heart Association,” Circulation 148, no. 24 (2023): 1982–2004, 10.1161/CIR.0000000000001191. 37947094

[6] N. A. ElSayed , G. Aleppo , V. R. Aroda , et al., “Classification and Diagnosis of Diabetes: Standards of Care in Diabetes—2023,” Diabetes Care 46, no. Supplement_1 (2023): S19–S40, 10.2337/dc23-S002. PMC9810477 36507649

[7] S. S. Khan , K. Matsushita , Y. Sang , et al., “Development and Validation of the American Heart Association's PREVENT Equations,” Circulation 149, no. 6 (2024): 430–449, 10.1161/CIRCULATIONAHA.123.067626. PMC10910659 37947085

[8] P. Muntner , D. Shimbo , R. M. Carey , et al., “Measurement of Blood Pressure in Humans: A Scientific Statement From the American Heart Association,” Hypertension 73, no. 5 (2019): e35–e66, 10.1161/HYP.0000000000000087. PMC11409525 30827125

[9] L. A. Inker , N. D. Eneanya , J. Coresh , et al., “New Creatinine‐ and Cystatin C–Based Equations to Estimate GFR Without Race,” New England Journal of Medicine 385, no. 19 (2021): 1737–1749, 10.1056/NEJMoa2102953. PMC8822996 34554658

[10] American Diabetes Association Professional Practice Committee for Diabetes , “Chronic Kidney Disease and Risk Management: Standards of Care in Diabetes—2026,” Diabetes Care 49, no. Supplement_1 (2026): S246–S260, 10.2337/dc26-S011. PMC12690176 41358881

[11] P. E. Stevens , S. B. Ahmed , J. J. Carrero , et al., “KDIGO 2024 Clinical Practice Guideline for the Evaluation and Management of Chronic Kidney Disease,” Kidney International 105, no. 4 (2024): S117–S314, 10.1016/j.kint.2023.10.018. 38490803

[12] Expert Panel on Detection, Evaluation, and Treatment of High Blood Cholesterol in Adults , “Executive Summary of the Third Report of the National Cholesterol Education Program (NCEP) Expert Panel on Detection, Evaluation, and Treatment of High Blood Cholesterol in Adults (Adult Treatment Panel III),” JAMA 285, no. 19 (2001): 2486–2497, 10.1001/jama.285.19.2486. 11368702

[13] K. G. M. M. Alberti , R. H. Eckel , S. M. Grundy , et al., “Harmonizing the Metabolic Syndrome: A Joint Interim Statement of the International Diabetes Federation Task Force on Epidemiology and Prevention; National Heart, Lung, and Blood Institute; American Heart Association; World Heart Federation; International Atherosclerosis Society; and International Association for the Study of Obesity,” Circulation 120, no. 16 (2009): 1640–1645, 10.1161/CIRCULATIONAHA.109.192644. 19805654

[14] D. M. Lloyd‐Jones , N. B. Allen , C. A. M. Anderson , et al., “Life's Essential 8: Updating and Enhancing the American Heart Association's Construct of Cardiovascular Health: A Presidential Advisory From the American Heart Association,” Circulation 146, no. 5 (2022): e18–e43, 10.1161/CIR.0000000000001078. PMC10503546 35766027

[15] WHO Expert Consultation , “Appropriate Body‐Mass Index for Asian Populations and Its Implications for Policy and Intervention Strategies,” Lancet 363, no. 9403 (2004): 157–163, 10.1016/S0140-6736(03)15268-3. 14726171

[16] Chronic Kidney Disease Prognosis Consortium , K. Matsushita , M. van der Velde , et al., “Association of Estimated Glomerular Filtration Rate and Albuminuria With All‐Cause and Cardiovascular Mortality in General Population Cohorts: A Collaborative Meta‐Analysis,” Lancet 375, no. 9731 (2010): 2073–2081, 10.1016/S0140-6736(10)60674-5. PMC3993088 20483451

[17] F. Mach , C. Baigent , A. L. Catapano , et al., “2019 ESC/EAS Guidelines for the Management of Dyslipidaemias: Lipid Modification to Reduce Cardiovascular Risk,” European Heart Journal 41, no. 1 (2020): 111–188, 10.1093/eurheartj/ehz455. 31504418

[18] T. Unger , C. Borghi , F. Charchar , et al., “2020 International Society of Hypertension Global Hypertension Practice Guidelines,” Hypertension 75, no. 6 (2020): 1334–1357, 10.1161/HYPERTENSIONAHA.120.15026. 32370572

[19] American Diabetes Association Professional Practice Committee for Diabetes , “Glycemic Goals, Hypoglycemia, and Hyperglycemic Crises: Standards of Care in Diabetes—2026,” Diabetes Care 49, no. Supplement_1 (2026): S132–S149, 10.2337/dc26-S006. PMC12690178 41358894

[20] S. A. Alfaraj , J. M. Kist , R. H. H. Groenwold , M. Spruit , D. Mook‐Kanamori , and R. C. Vos , “External Validation of SCORE2‐Diabetes in the Netherlands Across Various Socioeconomic Levels in Native‐Dutch and Non‐Dutch Populations,” European Journal of Preventive Cardiology 32, no. 7 (2025): 555–563, 10.1093/eurjpc/zwae354. 39485827

[21] H. Guo and H. Wu , “Comparison of Cardiovascular Risk Assessment Protocols and Achievement of Risk Factor Targets in Patients With Type 2 Diabetes,” Frontiers in Endocrinology 16 (2025): 1572202, 10.3389/fendo.2025.1572202. PMC12267027 40678314

[22] S. K. Low , C. F. Sum , L. Y. Yeoh , et al., “Prevalence of Chronic Kidney Disease in Adults With Type 2 Diabetes Mellitus,” Annals of the Academy of Medicine, Singapore 44, no. 5 (2015): 164–171, 10.47102/annals-acadmedsg.V44N5p164. 26198322

[23] P. Narindrarangkura , W. Bosl , R. Rangsin , and P. Hatthachote , “Prevalence of Dyslipidemia Associated With Complications in Diabetic Patients: A Nationwide Study in Thailand,” Lipids in Health and Disease 18, no. 1 (2019): 90, 10.1186/s12944-019-1034-3. PMC6451778 30954084

[24] D. Hyassat , S. Al‐Saeksaek , D. Naji , et al., “Dyslipidemia Among Patients With Type 2 Diabetes in Jordan: Prevalence, Pattern, and Associated Factors,” Frontiers in Public Health 10 (2022): 1002466, 10.3389/fpubh.2022.1002466. PMC9679519 36424970

[25] G. Hu , J. Ding , and D. H. Ryan , “Trends in Obesity Prevalence and Cardiometabolic Risk Factor Control in US Adults With Diabetes, 1999–2020,” Obesity 31, no. 3 (2023): 841–851, 10.1002/oby.23652. PMC9974736 36697975

[26] A. Misra , S. Anoop , and S. Sharma , “Obesity, Abdominal Obesity, and Adipose Tissue Depots in South Asians With Specific Reference to Asian Indians: Implications of New Obesity Guidelines,” Journal of Clinical Lipidology (2025), 10.1016/j.jacl.2025.06.012. 41293937

[27] A. D. Mooradian , “Dyslipidemia in Type 2 Diabetes Mellitus,” Nature Reviews. Endocrinology 5, no. 3 (2009): 150–159, 10.1038/ncpendmet1066. 19229235

