# Supplementary material for: Advanced Cardiovascular‐Kidney‐Metabolic Risk and Risk‐Factor Control in Patients With Type 2 Diabetes Without Established Cardiovascular Disease
Source: J Diabetes. 2026 Oct 19;18(10):e70279. doi: 10.1111/1753-0407.70279 (PMC13680869; doi:10.1111/1753-0407.70279)
Supplement: Supplementary file 1 [file JDB-18-e70279-s001.docx]

**SUPPLEMENTARY MATERIALS**

**TABLE OF CONTENTS**

[Supplementary Table S1. Triglyceride distribution of the study population	2](#_Toc231841304)

[Supplementary Table S2. Baseline characteristics according to advanced cardiovascular–kidney–metabolic risk status	3](#_Toc231841305)

[Supplementary Table S3. Center-level distribution of advanced CKM risk and risk-factor control	4](#_Toc231841306)

[Supplementary Table S4. Sensitivity analyses	5](#_Toc231841307)

[Supplementary Table S5. Blood pressure control according to the source of advanced cardiovascular–kidney–metabolic risk	7](#_Toc231841308)

[Supplementary Table S6. Site-clustering sensitivity analyses for risk-factor control outcomes	8](#_Toc231841309)

[Supplementary Table S7. Sensitivity analyses using risk-stratified lipid targets	10](#_Toc231841310)

[Supplementary Table S8. Exploratory sensitivity analysis using a modified PREVENT-derived classification after removing SBP-related terms	11](#_Toc231841311)

# **Supplementary Table S1. Triglyceride distribution of the study population**

***Panel A. Overall triglyceride distribution***

| **Triglyceride category** | **n/N (%)** |
| --- | --- |
| TG <1.7 mmol/L | 172 (12.0%) |
| TG ≥1.7 mmol/L | 1264 (88.0%) |
| TG >2.3 mmol/L | 1119 (77.9%) |
| TG >4.5 mmol/L | 701 (48.8%) |
| TG >11.3 mmol/L | 0 (0.0%) |

*Note. TG, triglycerides.*

***Panel B. Site-level triglyceride distribution***

| **Site** | **N** | **TG, median [IQR]** | **TG <1.7 mmol/L** | **TG ≥1.7 mmol/L** | **TG >2.3 mmol/L** | **TG >4.5 mmol/L** | **TG >11.3 mmol/L** |
| --- | --- | --- | --- | --- | --- | --- | --- |
| Hue University of Medicine and Pharmacy | 264 | 3.0 [1.8-4.8] | 60/264 (22.7%) | 204/264 (77.3%) | 167/264 (63.3%) | 81/264 (30.7%) | 0/264 (0.0%) |
| Thai Binh hospital | 255 | 4.8 [3.0-5.2] | 14/255 (5.5%) | 241/255 (94.5%) | 228/255 (89.4%) | 166/255 (65.1%) | 0/255 (0.0%) |
| Da Nang hospital | 216 | 3.2 [2.3-4.9] | 19/216 (8.8%) | 197/216 (91.2%) | 159/216 (73.6%) | 87/216 (40.3%) | 0/216 (0.0%) |
| Hue Central hospital | 206 | 3.8 [2.2-4.9] | 34/206 (16.5%) | 172/206 (83.5%) | 149/206 (72.3%) | 93/206 (45.1%) | 0/206 (0.0%) |
| Kien Giang General Hospital | 390 | 4.8 [3.0-5.3] | 40/390 (10.3%) | 350/390 (89.7%) | 321/390 (82.3%) | 203/390 (52.1%) | 0/390 (0.0%) |
| Nghe An Friendship General Hospital | 105 | 4.8 [3.0-5.3] | 5/105 (4.8%) | 100/105 (95.2%) | 95/105 (90.5%) | 71/105 (67.6%) | 0/105 (0.0%) |

*Note. Values are presented as median [interquartile range] or n/N (%). Abbreviation: TG, triglycerides; IQR, interquartile range.*

# **Supplementary Table S2. Baseline characteristics according to advanced cardiovascular–kidney–metabolic risk status**

| **Variable** | **Non-advanced CKM risk (n = 885)** | **Advanced CKM risk (n = 551)** | **P value** |
| --- | --- | --- | --- |
| Age, years | 57.0 [52.0-62.0] | 61.0 [56.0-66.0] | <0.001 |
| Female, n (%) | 429 (48.5%) | 252 (45.7%) | 0.312 |
| BMI, kg/m² | 23.0 [21.3-25.0] | 23.4 [21.4-25.6] | 0.072 |
| Duration of diabetes, years | 3.0 [2.0-5.0] | 3.0 [2.0-6.0] | 0.083 |
| Current smoker, n (%) | 191 (21.6%) | 175 (31.8%) | <0.001 |
| Using antihypertensive medication, n (%) | 499 (56.4%) | 419 (76.0%) | <0.001 |
| Using statins or lipid-lowering therapy, n (%) | 419 (47.3%) | 296 (53.7%) | 0.019 |
| Systolic blood pressure, mmHg | 120.0 [120.0-131.0] | 140.0 [120.0-150.0] | <0.001 |
| Diastolic blood pressure, mmHg | 70.0 [70.0-90.0] | 80.0 [70.0-100.0] | <0.001 |
| HbA1c, % | 7.7 [6.5-9.4] | 8.0 [6.7-9.2] | 0.286 |
| Total cholesterol, mmol/L | 4.9 [3.9-5.7] | 5.0 [3.9-6.0] | 0.022 |
| LDL-C, mmol/L | 2.9 [2.0-3.8] | 3.2 [2.1-4.3] | <0.001 |
| HDL-C, mmol/L | 1.0 [1.0-1.4] | 1.0 [0.9-1.2] | <0.001 |
| Non-HDL-C, mmol/L | 3.5 [2.7-4.4] | 3.9 [2.8-5.0] | <0.001 |
| Triglycerides, mmol/L | 4.7 [2.6-5.2] | 3.8 [2.5-4.9] | 0.229 |
| eGFR, mL/min/1.73 m² | 83.9 [69.7-96.1] | 55.7 [46.6-69.2] | <0.001 |
| PREVENT 10-year total CVD risk, % | 12.3 [8.7-15.7] | 22.6 [17.9-28.9] | <0.001 |

*Note. Values are presented as median [interquartile range] or n (%). Abbreviation: CKM, cardiovascular-kidney-metabolic; BMI, body mass index; HbA1c, glycated hemoglobin; LDL-C, low-density lipoprotein cholesterol; HDL-C, high-density lipoprotein cholesterol; eGFR, estimated glomerular filtration rate; CVD, cardiovascular disease.*

# **Supplementary Table S3. Center-level distribution of advanced CKM risk and risk-factor control**

| **Site** | **N** | **Advanced CKM primary** | **High PREVENT risk** | **eGFR <60** | **HbA1c control** | **BP control** | **LDL-C control** | **TG control** | **Non-HDL-C control** | **Combined lipid control** |
| --- | --- | --- | --- | --- | --- | --- | --- | --- | --- | --- |
| Kien Giang Hospital | 390 | 147/390 (37.7%) | 85/390 (21.8%) | 110/390 (28.2%) | 163/390 (41.8%) | 27/390 (6.9%) | 78/390 (20.0%) | 40/390 (10.3%) | 97/390 (24.9%) | 16/390 (4.1%) |
| Hue University of Medicine and Pharmacy Hospital | 264 | 83/264 (31.4%) | 69/264 (26.1%) | 46/264 (17.4%) | 45/264 (17.0%) | 129/264 (48.9%) | 37/264 (14.0%) | 60/264 (22.7%) | 35/264 (13.3%) | 8/264 (3.0%) |
| Thai Binh Hospital | 255 | 99/255 (38.8%) | 66/255 (25.9%) | 66/255 (25.9%) | 82/255 (32.2%) | 143/255 (56.1%) | 44/255 (17.3%) | 14/255 (5.5%) | 47/255 (18.4%) | 2/255 (0.8%) |
| Da Nang Hospital | 216 | 87/216 (40.3%) | 61/216 (28.2%) | 58/216 (26.9%) | 83/216 (38.4%) | 65/216 (30.1%) | 54/216 (25.0%) | 19/216 (8.8%) | 62/216 (28.7%) | 3/216 (1.4%) |
| Hue Central Hospital | 206 | 102/206 (49.5%) | 76/206 (36.9%) | 75/206 (36.4%) | 55/206 (26.7%) | 84/206 (40.8%) | 40/206 (19.4%) | 34/206 (16.5%) | 43/206 (20.9%) | 7/206 (3.4%) |
| Nghe An Friendship General Hospital | 105 | 33/105 (31.4%) | 24/105 (22.9%) | 15/105 (14.3%) | 50/105 (47.6%) | 33/105 (31.4%) | 17/105 (16.2%) | 5/105 (4.8%) | 19/105 (18.1%) | 4/105 (3.8%) |

*Abbreviation: BP, blood pressure; CKM, cardiovascular-kidney-metabolic; eGFR, estimated glomerular filtration rate; HbA1c, glycated hemoglobin; LDL-C, low-density lipoprotein cholesterol; TG, triglycerides; HDL-C, high-density lipoprotein cholesterol.*

# **Supplementary Table S4. Sensitivity analyses**

| **Outcome** | **Exposure** | **Adjusted OR (95% CI)** | **P value** |
| --- | --- | --- | --- |
| Glycemic control: HbA1c <7% | High PREVENT risk only | 0.795 (0.593-1.066) | 0.126 |
| Blood pressure control: age-specific ISH target |  | 0.407 (0.297-0.560) | <0.001 |
| LDL-C control: LDL-C <1.8 mmol/L |  | 0.668 (0.475-0.939) | 0.020 |
| Triglyceride control: TG <1.7 mmol/L |  | 0.722 (0.482-1.082) | 0.115 |
| Non-HDL-C control: non-HDL-C <2.6 mmol/L |  | 0.639 (0.461-0.886) | 0.007 |
| Combined lipid control: LDL-C, TG, and non-HDL-C |  | 0.447 (0.192-1.042) | 0.062 |
| Glycemic control: HbA1c <7% | Advanced CKM using PREVENT ≥20% or eGFR <45 | 0.769 (0.576-1.028) | 0.076 |
| Blood pressure control: age-specific ISH target |  | 0.431 (0.316-0.588) | <0.001 |
| LDL-C control: LDL-C <1.8 mmol/L |  | 0.688 (0.491-0.962) | 0.029 |
| Triglyceride control: TG <1.7 mmol/L |  | 0.734 (0.492-1.094) | 0.129 |
| Non-HDL-C control: non-HDL-C <2.6 mmol/L |  | 0.673 (0.488-0.928) | 0.016 |
| Combined lipid control: LDL-C, TG, and non-HDL-C |  | 0.439 (0.189-1.022) | 0.056 |
| Glycemic control: HbA1c <7% | Advanced CKM using PREVENT ≥20% or eGFR <30 | 0.790 (0.590-1.059) | 0.115 |
| Blood pressure control: age-specific ISH target |  | 0.417 (0.304-0.572) | <0.001 |
| LDL-C control: LDL-C <1.8 mmol/L |  | 0.686 (0.489-0.963) | 0.029 |
| Triglyceride control: TG <1.7 mmol/L |  | 0.720 (0.481-1.078) | 0.110 |
| Non-HDL-C control: non-HDL-C <2.6 mmol/L |  | 0.656 (0.474-0.907) | 0.011 |
| Combined lipid control: LDL-C, TG, and non-HDL-C |  | 0.447 (0.192-1.041) | 0.062 |
| Glycemic control: HbA1c <7% | Kidney-only: eGFR <60 | 0.833 (0.639-1.085) | 0.176 |
| Blood pressure control: age-specific ISH target |  | 0.618 (0.470-0.814) | <0.001 |
| LDL-C control: LDL-C <1.8 mmol/L |  | 1.312 (0.973-1.768) | 0.075 |
| Triglyceride control: TG <1.7 mmol/L |  | 0.813 (0.554-1.193) | 0.290 |
| Non-HDL-C control: non-HDL-C <2.6 mmol/L |  | 1.523 (1.146-2.024) | 0.004 |
| Combined lipid control: LDL-C, TG, and non-HDL-C |  | 1.323 (0.668-2.622) | 0.422 |

| Glycemic control: HbA1c <7% | Kidney-only: eGFR <45 | 0.600 (0.377-0.953) | 0.030 |
| --- | --- | --- | --- |
| Blood pressure control: age-specific ISH target |  | 0.917 (0.597-1.406) | 0.690 |
| LDL-C control: LDL-C <1.8 mmol/L |  | 1.498 (0.967-2.321) | 0.070 |
| Triglyceride control: TG <1.7 mmol/L |  | 1.113 (0.663-1.867) | 0.686 |
| Non-HDL-C control: non-HDL-C <2.6 mmol/L |  | 1.605 (1.055-2.442) | 0.027 |
| Combined lipid control: LDL-C, TG, and non-HDL-C |  | 0.944 (0.319-2.798) | 0.918 |
| LDL-C control: LDL-C <1.8 mmol/L | Advanced CKM primary, excluding TG >4.5 mmol/L | 0.876 (0.590-1.302) | 0.513 |
| Triglyceride control: TG <1.7 mmol/L |  | 0.618 (0.424-0.901) | 0.012 |
| Non-HDL-C control: non-HDL-C <2.6 mmol/L |  | 0.840 (0.590-1.197) | 0.335 |
| Combined lipid control: LDL-C, TG, and non-HDL-C |  | 0.772 (0.386-1.542) | 0.463 |
| BP control: age-specific target | Advanced CKM primary: PREVENT ≥20% or eGFR <60 | 0.466 (0.359-0.603) | <0.001 |
| BP control: uniform <130/80 mmHg |  | 0.421 (0.320-0.554) | <0.001 |
| BP control: uniform <140/90 mmHg |  | 0.467 (0.367-0.594) | <0.001 |

*Note. Adjusted models included age, sex, BMI, duration of diabetes, smoking status, and medication variables as appropriate for each outcome. Abbreviation: CKM, cardiovascular-kidney-metabolic; eGFR, estimated glomerular filtration rate; TG, triglycerides; BP, blood pressure; OR, odds ratio; CI, confidence interval.*

# **Supplementary Table S5. Blood pressure control according to the source of advanced cardiovascular–kidney–metabolic risk**

| **Advanced-risk source / overlap category** | **Blood pressure controlled, n/N (%)** |
| --- | --- |
| Cardiovascular-kidney-metabolic overlap | 45/200 (22.5%) |
| Cardiovascular-metabolic risk | 34/181 (18.8%) |
| Kidney-metabolic risk | 52/170 (30.6%) |
| Metabolic domain only | 350/885 (39.5%) |

# **Supplementary Table S6. Site-clustering sensitivity analyses for risk-factor control outcomes**

| **Sensitivity analysis** | **Outcome** | **Adjusted OR (95% CI)** | **P value** |
| --- | --- | --- | --- |
| Cluster-robust SE by hospital | Glycemic control: HbA1c <7% | 0.835 (0.572-1.221) | 0.353 |
|  | Blood pressure control: age-specific ISH target | 0.466 (0.325-0.667) | <0.001 |
|  | LDL-C control: LDL-C <1.8 mmol/L | 0.864 (0.696-1.074) | 0.188 |
|  | Triglyceride control: TG <1.7 mmol/L | 0.677 (0.432-1.060) | 0.088 |
|  | Non-HDL-C control: non-HDL-C <2.6 mmol/L | 0.922 (0.750-1.134) | 0.443 |
|  | Combined lipid control: LDL-C, TG, and non-HDL-C | 0.775 (0.523-1.149) | 0.205 |
| Cluster-robust SE by regions | Glycemic control: HbA1c <7% | 0.835 (0.533-1.310) | 0.434 |
|  | Blood pressure control: age-specific ISH target | 0.466 (0.289-0.750) | 0.002 |
|  | LDL-C control: LDL-C <1.8 mmol/L | 0.864 (0.770-0.971) | 0.014 |
|  | Triglyceride control: TG <1.7 mmol/L | 0.677 (0.452-1.014) | 0.058 |
|  | Non-HDL-C control: non-HDL-C <2.6 mmol/L | 0.922 (0.707-1.203) | 0.550 |
|  | Combined lipid control: LDL-C, TG, and non-HDL-C | 0.775 (0.312-1.925) | 0.583 |
| Hospital fixed-effect sensitivity | Glycemic control: HbA1c <7% | 0.821 (0.640-1.054) | 0.121 |
|  | Blood pressure control: age-specific ISH target | 0.412 (0.309-0.548) | <0.001 |
|  | LDL-C control: LDL-C <1.8 mmol/L | 0.824 (0.616-1.102) | 0.192 |
|  | Triglyceride control: TG <1.7 mmol/L | 0.712 (0.494-1.027) | 0.069 |
|  | Non-HDL-C control: non-HDL-C <2.6 mmol/L | 0.874 (0.662-1.155) | 0.344 |
|  | Combined lipid control: LDL-C, TG, and non-HDL-C | 0.795 (0.398-1.586) | 0.514 |
| Regions fixed-effect sensitivity | Glycemic control: HbA1c <7% | 0.835 (0.653-1.067) | 0.150 |
|  | Blood pressure control: age-specific ISH target | 0.417 (0.315-0.553) | <0.001 |
|  | LDL-C control: LDL-C <1.8 mmol/L | 0.865 (0.647-1.155) | 0.326 |
|  | Triglyceride control: TG <1.7 mmol/L | 0.690 (0.481-0.990) | 0.044 |
|  | Non-HDL-C control: non-HDL-C <2.6 mmol/L | 0.924 (0.701-1.218) | 0.574 |
|  | Combined lipid control: LDL-C, TG, and non-HDL-C | 0.767 (0.384-1.531) | 0.452 |

| Hospital random-intercept sensitivity | Glycemic control: HbA1c <7% | 0.822 (0.641-1.054) | 0.123 |
| --- | --- | --- | --- |
|  | Blood pressure control: age-specific ISH target | 0.413 (0.311-0.549) | <0.001 |
|  | LDL-C control: LDL-C <1.8 mmol/L | 0.856 (0.640-1.146) | 0.296 |
|  | Triglyceride control: TG <1.7 mmol/L | 0.710 (0.493-1.021) | 0.064 |
|  | Non-HDL-C control: non-HDL-C <2.6 mmol/L | 0.894 (0.677-1.180) | 0.428 |
|  | Combined lipid control: LDL-C, TG, and non-HDL-C | 0.778 (0.393-1.541) | 0.472 |

*Note. Models assessed the robustness of the association between advanced CKM risk and achievement of risk-factor control targets after accounting for hospital-level and regional clustering. Models used the same covariate structure as the primary adjusted analyses. Region-level cluster-robust models were considered exploratory because of the small number of regional clusters. Abbreviation: CKM, cardiovascular-kidney-metabolic; SE, standard error; LDL-C, low-density lipoprotein cholesterol; HDL-C, high-density lipoprotein cholesterol; TG, triglycerides; BP, blood pressure; OR, odds ratio; CI, confidence interval.*

# **Supplementary Table S7. Sensitivity analyses using risk-stratified lipid targets**

***Panel A. Risk-stratified lipid targets and risk-tier distribution***

| **Risk tier** | **N (%)** | **LDL-C target** | **non-HDL-C target** |
| --- | --- | --- | --- |
| Moderate risk | 51 (3.6%) | <2.6 mmol/L | <3.4 mmol/L |
| High risk | 941 (65.5%) | <1.8 mmol/L | <2.6 mmol/L |
| Very high risk | 444 (30.9%) | <1.4 mmol/L | <2.2 mmol/L |

*Note: According to the 2019 ESC/EAS Guidelines for the management of dyslipidaemias: lipid modification to reduce cardiovascular risk.*

***Panel B. Achievement of risk-stratified lipid targets according to advanced CKM risk status***

| **Outcome** | **Overall** | **Non-advanced CKM** | **Advanced CKM** | **P value** |
| --- | --- | --- | --- | --- |
| LDL-C control | 256/1436 (17.8%) | 186/885 (21.0%) | 70/551 (12.7%) | <0.001 |
| TG control | 172/1436 (12.0%) | 115/885 (13.0%) | 57/551 (10.3%) | 0.133 |
| non-HDL-C control | 287/1436 (20.0%) | 197/885 (22.3%) | 90/551 (16.3%) | 0.006 |
| Combined lipid control | 38/1436 (2.6%) | 29/885 (3.3%) | 9/551 (1.6%) | 0.059 |

***Panel C. Adjusted logistic regression using risk-stratified lipid targets***

| **Outcome** | **Adjusted OR for advanced CKM** | **P value** |
| --- | --- | --- |
| LDL-C control | 0.574 (0.421–0.782) | <0.001 |
| TG control | 0.677 (0.473–0.969) | 0.033 |
| non-HDL-C control | 0.697 (0.523–0.930) | 0.014 |
| Combined lipid control | 0.469 (0.214–1.027) | 0.058 |

# **Supplementary Table S8. Exploratory sensitivity analysis using a modified PREVENT-derived classification after removing SBP-related terms**

***Panel A. Distribution of modified PREVENT-derived risk classifications***

| **Classification** | **n/N (%)** |
| --- | --- |
| High PREVENT risk ≥20%, original PREVENT column | 381/1436 (26.5%) |
| High PREVENT risk ≥20%, exploratory PREVENT after removing SBP-related terms | 310/1436 (21.6%) |
| Advanced CKM, original PREVENT ≥20% or eGFR <60 | 551/1436 (38.4%) |
| Advanced CKM, exploratory PREVENT without SBP-related terms ≥20% or eGFR <60 | 492/1436 (34.3%) |
| Advanced CKM, exploratory PREVENT without SBP-related terms ≥20% or eGFR <45 | 321/1436 (22.4%) |
| Advanced CKM, exploratory PREVENT without SBP-related terms ≥20% or eGFR <30 | 311/1436 (21.7%) |

***Panel B. Risk-factor control according to modified advanced CKM status***

| **Outcome** | **Non-advanced CKM** | **Advanced CKM** |
| --- | --- | --- |
| Glycemic control | 337/944 (35.7%) | 141/492 (28.7%) |
| BP control | 341/944 (36.1%) | 140/492 (28.5%) |
| LDL-C control | 180/944 (19.1%) | 90/492 (18.3%) |
| TG control | 125/944 (13.2%) | 47/492 (9.6%) |
| Non-HDL-C control | 195/944 (20.7%) | 108/492 (22.0%) |
| Combined lipid control | 26/944 (2.8%) | 14/492 (2.8%) |

***Panel C. Adjusted logistic regression using modified advanced CKM status***

| **Outcome** | **Adjusted OR** | **P value** |
| --- | --- | --- |
| Glycemic control | 0.731 (0.569–0.940) | 0.015 |
| Blood pressure control | 0.722 (0.560–0.932) | 0.012 |
| LDL-C control | 0.923 (0.689–1.236) | 0.591 |
| TG control | 0.607 (0.417–0.885) | 0.009 |
| Non-HDL-C control | 1.037 (0.786–1.369) | 0.798 |
| Combined lipid control | 0.838 (0.418–1.678) | 0.618 |

*Note. In this exploratory sensitivity analysis, all SBP-related components were removed from the original sex-specific PREVENT linear predictor. Specifically, the omitted terms were the cSBP and cSBP2 main terms, the cSBP2 × antihypertensive treatment interaction, and the cAge × cSBP2 interaction, where cAge = (age − 55)/10, cSBP = [min(SBP, 110) − 110]/20, and cSBP2 = [max(SBP, 110) − 130]/20. The main effect of antihypertensive treatment and all other terms were retained with their original coefficients. No coefficients were re-estimated or recalibrated. This modified PREVENT-derived classification was used solely as an exploratory sensitivity analysis and does not represent a validated alternative PREVENT equation.*
